# Does Past Experience Justify Continued Use of Cohesive Gold

**Published:** 1875-08

**Authors:** A. O. Rawls


### DOES PAST EXPERIENCE JUSTIFY CONTINUED USE OF COHESIVE GOLD.

BY A. O. RAWLS, D. D. S.

*Read before Kentucky State Association.*

It is evidently as true of cohesive foil as with other materials for filling teeth, that it has met approval very much in proportion to the amount of honest investigation of its qualities, or accordingly as its merits are viewed with an eye to success in this or that direction. Thus while the observations of some have led them to deprecate, and condemn its use for the reason that as a standard filling in a certain class of cavities it had not equaled their expectations. Others have been pleased to accord it merit and encourage its use from the fact that in their practice and that of their co-laborers, they have been able to see much good result, aside from the foregoing or any special view of its applications. And in presenting this subject for your consideration I wish not to be understood as favoring its claims for any single virtue it may possess, but rather for the reason of its usefulness in general, for its adaptation to every class of work wherein other forms of gold are used for filling teeth, and also because of the most excellent effects of its introduction on the practice of operative dentistry throughout. Should the question be asked has cohesive foil under all circumstances, in the hands of those attempting its use, proved of good effect, I would certainly answer in the negative, but the question as it is refered to this society for consideration, I am fully persuaded that my reply should be in the affirmative, indeed, did I not thus respond, and that with as good grace as ever man wedged a cylinder of non. cohesive gold, I would surely be recreant to the cause I willingly espouse. In noting the various changes wrought from time to time in the development of science or art, in political and social government or, in fact, in all the many progressive systems of the world's history, there is no feature more prominent in a radical revolution of any of these systems, than that of determined opposition to its accomplishment, a reluctance to giving up old things for fear that in accepting the new failure may result. And though it may be truly said of opposition that as a rule it exerts a healthful influnce on the progressive development of our interests, yet it is of equal truth that there comes a time when this ultra spirit of antagonism would be very much out of place, except wherein it opposed real and not imaginary discrepancies. The growth of cohesive foil to its present status in dentistry, has not been without a struggle.

In the hands of not a few it has proved intractable, and of very uncertain merit, while in the practice of many in fact in the main, when it has been employed with the same determination to succeed that has characterized skillful hands in the use of non-cohesive gold, it has not only established a value equal to the best, but in very many cases has surpassed in necessary requirements every form of material, designed for filling teeth.

As in the consideration of a statement we may err in our judgment, unless the mind is brought to bear on the subject matter comparatively free from existing predilections, so also we may fail to secure many things of benefit in the discussion of a subject unless we can for a time freely lay aside our prejudices and be ready to recieve the truth when established. And now let us inquire as to what should constitute our aim in filling teeth. This may appear a very simple inquiry, and yet, judging from the different ideas entertained on the subject of success, as it pertains to filling teeth, we are constrained to make the same. We are aware that in this as in any good undertaking our aim should be success, nevertheless this answer, if given from different standpoints, may or may not be correct, true success can not be attained in this direction without there be an appropriate estimation as to the requirements, and probale results of an operation. For instance, when we see fillings that have been in the teeth for several years, we are wont to say of them they are successful operations, and usually in this our judgment is predicted on the fact of said fillings being in situ, preventing further decay in their respective places, and indeed presenting an appearance much the same as when first placed there. But to pass judgment thus on all such fillings as have retained their integrity for considerable time, disregarding other, and just as essential requisites to success, would evidently be looking at the case from an incorrect standpoint. In our efforts to perfect the art of filling teeth, it is clearly of importance, that our aim should be not only to so complete the work that there may be favorable chances for its retention, but also in a maner that will undoubtedly seem to the patient the most effective results, as to use and comfort. We recognize, in not a few cases coming to our notice, the many annoying difficulties tending to prevent successful consummation of our labor, and yet we verily belive that some of these difficulties might be overcome did our profession possess more skill in the manipulation of all material, and appliances, now extant for the purpose.

Touching the filling or teeth such as in their preparation require no material destruction of their general contour, it matters not, or but little, whether we use cohesive or non-cohesive gold, so it be good of its kind, and fitly applied, but in order that we may thoroughly understand and appreciate the merits of different substances or different forms of the same material for replacing broken down tooth structure, we should by all means subject them to tests wherein all things else would be equal, not merely in ordinary cavities of decay nor in teeth, the structure of which exhibited more favorable characteristics in behalf of one than of the other material, but also in cases most complex, as they refer to size of the cavity, difficulty of reaching the same, constitution, temperament and peculiar idiosyncrasies of the patient. In fact the existence of anything which might in any respect have bearing on the results of such operations should be duly considered.

It would doubtless be well to make the application of these comparative tests more especially to the latter class of cavities, since to this complex variety our failures are mostly ascribed. We are happily possessed of ample material and means for producing successful results in the former, ordinary class of fillings, but to make the most of desperate cases is often truly perplexing, in consequence of which they really deserve more special attention; indeed if we would be a progressive profession our labor must of necessity lead us away from things of easy accomplishment and into the more abstruse problems of our calling. To lay aside however, the probability of any tests being made, under conditions above mentioned and to consult the experience of very many of our best operators, who have in the use of cohesive foil made it a thorough study, and its application to this class of work worthy their best efforts, we verily believe that deductions from a past comparison of such experience with similar experience in the employment of any other form of gold, would without doubt greatly favor the superiority of cohesive gold.

It is a matter of no little surprise that men who pretend to seek the truth, urge so many objections against the use of a material, the nature of which they have never yet cared to ascertain, and as objections often tend to confirm the truth, we trust that in vindication of our position we may be granted the right of mentioning a few of the more prominent ones.

Among the first objections greeting our ears was difficulty in manipulating cohesive gold on account of its tendency to roll up or clog under the instrument, which to some extent was true at the time it was first urged, but to use that in argument against it to-day would, to say the least, be severely absurd. Parsimonious investigatiors for a time grew exceedingly wroth and regreted very much that it required such waste of material. Also it has been urged that this class of work would not stand. If this be true then indeed will the labor of no inconsiderable number of our best operators have been in vain, but its concontinual use and the fact that such work has stood well from the time its peculiarities were undestood is a sufficient guarantee of its virtues in that direction, and really a positive denial of the foregoing objection.

Again it has been said that it could not be adapted as perfectly to the walls of a cavity as could non-cohesive gold, and among the reasons given why this could not be done was that of its non elasticity. Now let us see wherein elasticity would be of benefit, or if you please the philosophy of an elastic property, as it would enhance the merits of any material for filling teeth. To accept a common sense view of the matter, the only philosophic reason which could be urged favoring the elastic property of gold as a filling, would be that of its tendency after compression to spring out from center to circumference, to adapt itself to the walls of a cavity more completely than could the force ordinarily used for that purpose. If it be true of any kind of gold in use, that it has sufficient elasticity to reclaim its position upon removal of the force which displaced it, then the aforesaid reason might be relevant. If not, however, then the elastic condition, if such existed, of the material would under pressure used for its adaptation speedily resolve itself into that of a passive nature in the which, so far as placing it in contact with the walls of the cavity is concerned, would be of no more benefit than lead or any non-elastic substance. In a word this property would virtually be of no avail when destroyed by the force of its application.

Another objection, and one which should have been accredited to the operator rather than, as it has been, to the peculiarities of the gold, is that in making additions, piece by piece and malleting each successive particle there existed a continual disturbing of the mass beneath, a tendency to displace the adaptation of preceeding portions of the fillings. This only holds good with the proviso that its reference be to the exception and not the rule, to a lack of skillful manipulation or an oversight in securing proper anchorage, rather than to the supposed impossibillity of the latter. In truth if cohesive gold be used with an appropriate appreciation of its peculiarities, a thorough knowledge of how it should be used and with instruments best suited to the work, all disturbance of its particles will be as clearly in the right direction as would block or cylinder of non-cohesive foil under the hands of an operator skilled in its use.

To the objections that it consumes too much time to properly complete an operation after this method and that it is of such necessary importance that the cavity, gold and instruments be absolutely dry. We will of the former contend that the difference of time requisite in the use of the one or other form of gold, when applied to ordinary sized cavities is of but little or no concern. While for larger fillings it may, perhaps does, take more time, but in as much as it does we hope to arrive at better results and to have corresponding increase of benefit, accrue to the recipients of our labor.

To the latter of the foregoing objections, we can not do better, in order that we may refute the fallacy of such argument, than to solicit a consideration of the reasons why the arrest of decay is accomplished by excavating and filling perfectly the decayed cavity. Further comment we deem unnecessary.

Much also has been said in condemnation of this practice for the supposed reason that it was necessary to so shape the work as to present more gold to view than would be the case with non-cohesive gold. To accept this however as it is usually applied, would certainly be of doubtful policy, for it is a statement void of plausible grounds of defence. All ordinary work of this character can be as completely hidden from view and with as much ease of effort as can similar work by any means resorted to. Though if such complaints be referred to that class of fillings usually styled contour and especially when they are large and situated in the anterior teeth. Then anything said pro or con on the subject would of necessity lead us into a consideration of the merits or demerits of contour filling, in general, which is a subject in itself, and had better be discussed under that particular head.

I have made mention of the fact that since the introduction of cohesive gold it had proved of excellent effect on the practice of operative dentistry in general, and in making such statement I was not influenced alone by a knowledge of its merits as a material for filling teeth, but aside from this consideration, it was my desire to refer more especially to the fact that its introduction had given an impetus to varied interests of the profession, in the which very much of benefit had fallen to ourselves and our patients.

Very soon after its introduction, the necessity for a complete freedom from moisture in its manipulation, was ascertained, and not many year's thereafter our profession was greeted with one of the grandest, most useful anxiliaries to operative dentistry ever presented, Barnum's Rubber Dam.

Many appliances and attachments to various kinds of dental burring engines have been called into existence, purely as the result of demands coming from the use of cohesive gold. The improvement if not origin of gold screws as a means for better securing fillings of a certain class is surely one of the valuable outgrowths of this practice, and although these improvements have been and are now of such value to that practice which has brought them into use, it does not follow that they are of no account to those employing cohesive gold in their practice, but on the contrary, there is not a single one of these but what can be made to serve a good purpose, no matter what the mode of operating. It has been the means of arousing the latent energies of its numerous devotees, and stimulating into renewed action, those who so fondly clung to the old regime. Before its use there existed but one form of recognized standard work; consequently not so much apparent necessity for emulative spirit, but from the moment cohesive gold began to contend with non-cohesive for supremacy, the efforts on the part of the faithful in behalf of their respective modes of practice has been redoubled with a view that smacked of future good, and I firmly believe as a result, we are approaching true success, though by different routes, nevertheless just as surely.

The extremes have been reached, many things which were of doubtful practice, have gone into disuse and I trust we are rapidly and gracefully leveling up and leveling down and may ere long reach the always sought for, golden mean.

